# Newly characterised *ex vivo* colospheres as a three-dimensional colon cancer cell model of tumour aggressiveness

**DOI:** 10.1038/sj.bjc.6605173

**Published:** 2009-07-14

**Authors:** L-B Weiswald, S Richon, P Validire, M Briffod, R Lai-Kuen, F P Cordelières, F Bertrand, D Dargere, G Massonnet, E Marangoni, B Gayet, M Pocard, I Bieche, M-F Poupon, D Bellet, V Dangles-Marie

**Affiliations:** 1 IFR71 Sciences du Médicament, Faculté des Sciences Pharmaceutiques et Biologiques, Université Paris Descartes, 4 avenue de l’Observatoire, F-75006 Paris, France; 2 Département d’Anatomie Pathologique, Institut Mutualiste Montsouris, 42 boulevard Jourdan, F-75014 Paris, France; 3 Service d’Anatomie et de Cytologie Pathologiques, Centre René Huguenin, 35 rue Dailly, F-92210 Saint Cloud, France; 4 Plateforme d’Imagerie Cellulaire et Moléculaire, IFR71 Sciences du Médicament, Faculté des Sciences Pharmaceutiques et Biologiques, Université Paris Descartes, 4 avenue de l’Observatoire, F-75006 Paris, France; 5 Plateforme Imagerie Cellulaire et Tissulaire, Research Center, Institut Curie, 26 rue d’Ulm, F-75005 Paris, France; 6 Département du Transfert, Hôpital Institut Curie, 26 rue d’Ulm, F-75005 Paris, France; 7 Département Médico-Chirurgical de Pathologie Digestive, Institut Mutualiste Montsouris, 42 boulevard Jourdan, F-75014 Paris, France; 8 Département Médico-Chirurgical de Pathologie Digestive Chirurgie, Hôpital Lariboisière, 2 rue Ambroise Paré, F-75010 Paris, France; 9 UMR U965 INSERM/Paris7 Université Paris Diderot, Hôpital Lariboisière, F-75010 Paris, France; 10 UMR745 INSERM, Faculté des Sciences Pharmaceutiques et Biologiques, Université Paris Descartes, 4 avenue de l’Observatoire, F-75006 Paris, France; 11 Service de Médecine Nucléaire, Centre René Huguenin, 35 rue Dailly, F92210 Saint Cloud, France

**Keywords:** colospheres, colorectal cancer, three dimension, *ex vivo* model, metastasis

## Abstract

**Background:**

New models continue to be required to improve our understanding of colorectal cancer progression. To this aim, we characterised in this study a three-dimensional multicellular tumour model that we named colospheres, directly obtained from mechanically dissociated colonic primary tumours and correlated with metastatic potential.

**Methods:**

Colorectal primary tumours (*n*=203) and 120 paired non-tumoral colon mucosa were mechanically disaggregated into small fragments for short-term cultures. Features of tumours producing colospheres were analysed. Further characterisation was performed using colospheres, generated from a human colon cancer xenograft, and spheroids, formed on agarose by the paired cancer cell lines.

**Results:**

Colospheres, exclusively formed by viable cancer cells, were obtained in only 1 day from 98 tumours (47%). Inversely, non-tumoral colonic mucosa never generated colospheres. Colosphere-forming capacity was statistically significantly associated with tumour aggressiveness, according to AJCC stage analysis. Despite a close morphology, colospheres displayed higher invasivity than did spheroids. Spheroids and colospheres migrated into Matrigel but matrix metalloproteinase (MMP)-2 and MMP-9 activity was detected only in colospheres. Mouse subrenal capsule assay revealed the unique tumorigenic and metastatic phenotype of colospheres. Moreover, colospheres and parental xenograft reproduced similar CD44 and CD133 expressions in which CD44^+^ cells represented a minority subset of the CD133^+^ population.

**Conclusion:**

The present colospheres provide an *ex vivo* three-dimensional model, potentially useful for studying metastatic process.

Colorectal cancer (CRC) is a major cause of morbidity and mortality worldwide, and CRC patient death is generally attributable to metastasis development and resistance to chemotherapy. Human CRC is one of the most extensively investigated tumour types, and genetic pathways involved in these malignancies have been identified (Fearon and Vogelstein, 1990). However, complex factors involved in CRC metastasis remain largely undefined and there is a need for effective drugs for treatment. In this context, successful future treatments must rely on a comprehensive analysis of events underlying the metastatic process, together with the development of new model systems that could be easily manipulated so as to evaluate the efficacy of novel therapeutics. The importance of studying cancer cells in 3D has been recently emphasised because of their higher relevance for an *in vivo* situation (Jacks and Weinberg, 2002; O’Brien *et al*, 2002; Yamada and Clark, 2002; Debnath and Brugge, 2005; Smalley *et al*, 2006). For this purpose, some permanent cancer cell lines, including colon cancer cell lines, are able to form spheroids when cultured in non-adherent conditions (for review, Friedrich *et al*, 2007). These spheroids are recognised to mimic microtumours more closely than cancer cell line monolayers and have been used mainly in chemo- and radioresistance studies. Another 3D cell model aims at promoting the expansion of cancer stem cells from solid tumour tissue after enzymatic dissociation in single-cell suspensions. This approach, first described for the expansion of normal or cancer brain cell in neurospheres (Svendsen *et al*, 1998), required a specific culture protocol and, in the case of CRC, led to the colon cancer sphere model (Ricci-Vitiani *et al*, 2007; Todaro *et al*, 2007; Vermeulen *et al*, 2008).

In this study, carried out on a series of 203 CRC primary tumours, we describe a model of 3D cellular structures spontaneously generated after an *ex vivo* mechanical dissociation of CRC tissue. We refer to these 3D structures as colospheres. To further characterise these colospheres, we used a human colon cancer xenograft, XenoCT320, and the derived paired colon cancer cell lines, CT320X6 and CT320 (Dangles-Marie *et al*, 2007). Colospheres generated from xenograft tissue and spheroids formed on agarose from colon cancer cell lines were then studied in parallel.

## Materials and methods

### Patients and tissue specimens

The CRC primary tumours were collected from the Institut Mutualiste Montsouris (Paris, France) and the Institut Gustave Roussy (Villejuif, France) from 203 patients observed between May 2003 and May 2006, in accordance with protocols approved by the local ethical committees and with the Helsinki Declaration of 1975, as revised in 1983.

Tumour material not required for histopathological diagnosis was placed in a ‘culture medium’ (DMEM supplemented with 10% decomplemented FCS, 10 mmol l^−1^ HEPES, 4.5 g l^−1^ glucose, 1 mmol l^−1^ pyruvate sodium, 200 U ml^−1^ penicillin and 200 *μ*g ml^−1^ streptomycin) and submitted to *ex vivo* mechanical mincing.

### Colosphere-forming assay

Tissue fragments of approximately 0.5 × 0.5 × 0.5 cm were submitted to mechanical dissociation in Petri dishes: after a medium washing step, tissue sample was first finely cut with a scalpel blade and then crushed with a striated plunger from a disposable syringe. The resulting pieces were transferred to a 25-cm^2^ culture flask containing the ‘culture medium’ and incubated at 37°C, in 8% CO_2_.

Dissociated tumour cultures were scored by a double-blind light microscopical examination at × 10 magnification by two different observers. A minimum of five fields per flask were viewed. Colosphere formation was scored on day 1 after culture as follows: (−), no colosphere observed; (+), ⩽2 colospheres per field; (++), >2 colospheres per field.

### Xenograft and spheroid culture

XenoCT320 xenograft, CT320 and CT320X6 cell lines have been previously described (Dangles-Marie *et al*, 2007). For xenograft passage, tumour fragments were subcutaneously grafted in the interscapular region into 5-week-old athymic nude female mice (Harlan, Winkelmann, Germany) bred and maintained in specified pathogen-free conditions (protocol approval no P2.VDM.026.07, local ethical committee on animal experiments, CREEA René Descartes, Paris, France).

For spheroid culture, tumour cells grown as a monolayer were resuspended with trypsin, and 5 × 10^3^ cells were seeded in microwells coated with 1% agarose so as to obtain, after 3 days, a single spheroid per well.

### 2D multipositioning light videomicroscopy

Colosphere and spheroid development was monitored by time-lapse video microscopy for 65 h at a 4 min interval. Dynamic sequences were obtained on a DM IRBE stand equipped with a motorised stage (Leica, Mannheim, Germany) using a 37°C 8% CO_2_-humidified stage-top incubator (Life Imaging Services, Basel, Switzerland).

### Histological characterisation

Colospheres and spheroids were embedded using the Cytoblock method (Briffod *et al*, 2000) and the Shandon kit (Thermo electron corporation, Saint Herblay, France). Immunostaining was performed on the resulting paraffin sections using an automated immunostainer (Ventana, Strasbourg, France) with mAb to Ki67 antigen (MIB1 clone; Dako, Trappes, France) and to E-cadherin (4A2C7 clone; Zymed, Montrouge, France). At least eight independent samples were collected for the specific analysis of colospheres and spheroids.

### *TBP* gene expression

Specific mouse *TBP* gene expression and the expression of both the mouse and the human *TBP* genes were studied by real-time quantitative RT–PCR (Lévy *et al*, 2004) to determine the quantity of mouse cells in human xenografts and colospheres. With the assistance of the computer program Oligo 5.0 (National Biosciences, Plymouth, MN, USA), the murine *Tbp* primer pair was selected to be mouse specific when compared with the sequence of the human *TBP* gene, whereas the total *TBP* primer pair was selected to amplify both the mouse and the human *TBP* genes. BLASTN searches against dbEST and nr (the non-redundant set of the GenBank, EMBL and DDBJ database sequences) were conducted to confirm the total gene specificity of the nucleotide sequences chosen for the primers and for the absence of DNA polymorphisms. The nucleotide sequences of the primers used were the following: Mm-TBP-U (5′-CCCTTGTACCCTTCACCAATGAC-3′) and Mm-TBP-L (5′-TCACGGTAGATACAATATTTTGAAGCTG-3′) and Total-TBP-U (5′-TGCACAGGAGCCAAGAGTGAA-3′) and Total-TBP-L (5′-CACATCACAGCTCCCCACCA-3′). The thermal cycling conditions comprised an initial denaturation step at 95°C for 10 min, and 50 cycles at 95°C for 15 s and 65°C for 1 min.

Results, expressed as *N*-fold differences in the specific murine *Tbp* gene expression (using Mm-TBP primers), relative to the sum of the mouse and the human *TBP* gene expression (using Total-TBP primers), termed N*Mm-TBP*, are determined by the formula: N*Mm-TBP*=2^Δ*C*tsample^. The Δ*C*_t_ value of the sample is determined by subtracting the *C*_t_ value of the murine *TBP* gene from the *C*_t_ value of the total (mouse+human) *TBP* gene. The N*Mm-TBP* values of the samples are subsequently normalised such that the median of the N*Mm-TBP* values of four mouse tissues was 100. As *TBP* is a ubiquitously expressed housekeeping gene, which encodes the TATA box-binding protein, a component of the DNA-binding protein complex TFIID, and shows a similar expression in our human and mouse tissues (*C*_t_=27 for 5 ng cDNA), the final result (normalised N*Mm-TBP* value) determines the proportion of mouse cell contamination for a given sample.

### Electron microscopy

After harvesting, colospheres and spheroids were analysed using standard techniques for transmission electron microscopy (Ribadeau Dumas *et al*, 2004). Ultrathin sections were examined at 80 kV using a JEOL JEM-1005 electron microscope (JEOL S.A., Croissy-sur-Seine, France). For scanning electron microscopy (SEM), cells were critical-point dried in hexamethyldisilizane (Sigma-Aldrich, Saint-Quentin, France). Samples were mounted on specimen stubs, sputter coated with gold using JEOL JCF100 and examined at 16 kV using JEOL ISM-35CF. At least six samples for CT320X6 and CT320 spheroids and for XenoCT320 colospheres were submitted to electron microscopy examination.

### *In vivo* tumorigenicity assay

The tumorigenicity of XenoCT320 colospheres, CT320X6 spheroids and CT320X6 single cells was compared in a subrenal capsule assay in nude mice. Three days after xenograft tissue dissociation, colospheres of 100–150 *μ*m diameter were manually collected under the microscope and an aliquot was trypsinised to estimate the number of viable cells in the colospheres. The concentration was adjusted to have a number of colospheres equivalent to 4 × 10^4^ cells per 10 *μ*l for intrarenal injection. Similarly, eight spheroids formed by seeding 5 × 10^3^ cells per microwell were injected in 10 *μ*l. As spheroids from this cell line grow slowly, spheroids collected 3 days after initiation contained about 5000 cells per spheroid for a diameter of about 150 *μ*m. An oblique incision was made on the skin parallel to the long axis of the right kidney in anaesthetised mice (xylazin/ketamin protocol). Injections of cells equivalent to 4 × 10^4^ cells were administered with a 27 G needle in the subcapsular space in the right kidney. The internal diameter of the 27 G needles is 200 *μ*m at the minimum, which would avoid any damage on spheroids and colospheres. After cell injection, the kidney was then returned to the peritoneal space and the skin was closed with surgical staples. The recipient mice were necropsied at 14 weeks after injection and kidneys, spleen, lung and liver were removed for histological examination.

Tumorigenicity of the CT320X6 cell line was carried out in parallel in nude mice by the subcutaneous transplantation of 2 × 10^6^ viable cells in 0.2 ml of PBS. The mice were observed for 2 months for the appearance of tumour development.

### Matrigel assay

Colospheres and spheroids were added to Matrigel solution (3 mg ml^−1^) (BD Biosciences, Le Pont de Claix, France) in 24-well plates before polymerisation of the lattice. Culture medium was added to the Matrigel layer.

### Gelatin zymography

Gelatin zymography assays were performed with a Bio-Rad laboratories kit (Marnes La Coquette, France), according to the manufacturer's instructions. Colospheres, spheroids and xenograft tissue were lysed with zymogram sample buffer and the amount of protein was estimated using the Bradford method (Biorad Dc Protein Assay; Bio-Rad Laboratories). Twenty micrograms of protein from each sample was separated on polyacrylamide gels containing 10% gelatin. After electrophoresis, the gels were washed in renaturation buffer for 30 min, and incubated overnight at 37°C in the development buffer. Gels were stained with a Coomassie blue R250 0.5% solution and destained with 3 × destaining solution.

### Flow cytometric analysis

Disaggregated colospheres, spheroids and xenografts were assessed using an Epics XL cytometre (Coulter, Villepinte, France) with the following antibodies: anti-human CD133/2 (clone 293C3) phycoerythrin, anti-human EpCAM (clone HEA-125) fluorescein isothiocyanate (Miltenyi-Biotec SAS, Paris, France) and anti-human CD44-FITC (clone G44-26; BD Biosciences), at appropriate dilutions. As for xenograft tissue, human colon cancer cells were magnetically labelled and separated from mouse stroma cells using human EpCAM microbeads (Miltenyi-Biotec SAS) according to the manufacturer's instructions, before assessment by flow cytometry.

### Confocal microscopy

About 30 colospheres in suspension were fixed for 3 h at 4°C in PBS containing 4% PFA, followed by washes in PBS. Colospheres were then permeabilised in 1% Triton X-100 in PBS for 1 h and washed with PBS. After a 1 h incubation in PBS/NH_4_Cl (50 mmol l^−1^), followed by washes in PBS, colospheres were saturated overnight with 3% BSA in PBSt (0.1% Triton X-100 in PBS) at 4°C and washed in PBSt. Colospheres were incubated for 24 h at 4°C with anti-human EpCAM-FITC (clone HEA-125) in PBSt (dilution 1 : 50) and rinsed with PBSt. The DNA marker, TOPRO-3 (Invitrogen-Molecular Probes, Cergy Pontoise, France), was then applied for 20 min at room temperature. Colospheres were mounted in glycerol/PBS (90/10: v/v). Images were recorded on a Leica TCS SP2 confocal microscope.

## Results

### Observation of colospheres within *ex vivo* dissociated colon primary tumours

**Figure 1:**
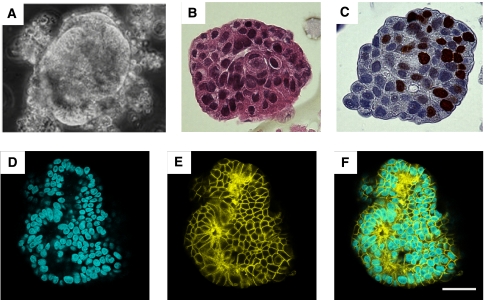
Colospheres derived from colon carcinoma patient tissue. (**A**) Photomicrograph of a representative colosphere generated on tissue culture plastic; (**B**) HES staining; (**C**) anti-Ki67 staining. Magnification: × 40. (**D–F**) Confocal immunofluorescence of colospheres. (**D**) TOPRO-3 staining; (**E**) EpCAM staining; (**F**) overlay of (**D**) and (**E**). Scale bar: 50 *μ*m.

Dissociation of 203 fresh colon primary tumour tissues directly harvested from human patients undergoing surgery led to the formation of spherical organoid structures, which we named colospheres (Figure 1A), in 98 out of the 203 specimens (47%) in just 1 day. Colospheres displayed a diameter comprised between 20 and 200 *μ*m and were easily identifiable because of their smooth refringent outline. To determine whether the property to form such colospheres from patient tumours was a feature restricted to cancer tissue, the same mechanical dissociation protocol was carried out in parallel with non-tumoral colon tissue counterparts from 120 of these 203 tumour tissues. It is noteworthy that no colospheres were ever observed after dissociation of non-neoplastic colonic mucosa, showing that these spherical structures are only generated by cancer tissue. Among these 98 specimens, 28 tumours led to the generation of a large number of colospheres.

Colospheres formed by highly compacted cells were resistant to mechanical disruption. On the basis of haemalun–eosin–safran (HES) staining, only carcinoma cells were observed in colospheres, whereas no stromal cells were recognised (Figure 1B), indicating that colospheres were structured aggregates of cancer cells and not merely globular fragments of tumour tissue. EpCAM staining performed with cytometry analyses or confocal microscopy confirmed the epithelial origin of colosphere-forming cells (Figure 1D–F). Anti-Ki67 immunostaining showed that colospheres contained viable proliferating cells (Figure 1C).

**Figure 2:**
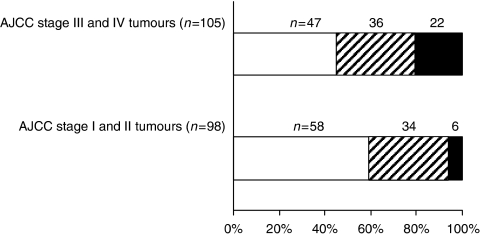
Colosphere formation is associated with tumour aggressiveness. Figure showing the number of primary tumours giving rise to many colospheres (‘++’, ▪), a few colospheres (‘+’,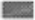) or no colosphere (‘−’, □) in non-disseminated tumours (AJCC stages I and II) and in disseminated tumours (AJCC stages III and IV). Colosphere formation was scored as described in Materials and Methods.

Tumour collection was divided into two groups: non-disseminated tumours (AJCC stage I and II) and disseminated tumours (AJCC stage III and IV), as AJCC stage I and II tumours differ from stage III and IV tumours by the fact that the latter group displays metastases in lymph nodes and/or in distant organs. Thus, the capacity of primary tumours to form colospheres was found to be significantly correlated with tumour aggressiveness. Indeed, statistical analysis showed that the stage III and IV tumour groups gave rise more frequently to colospheres than did the stage I and II tumour groups (*P*<0.01; Figure 2).

### Colosphere morphology studies

**Figure 3:**
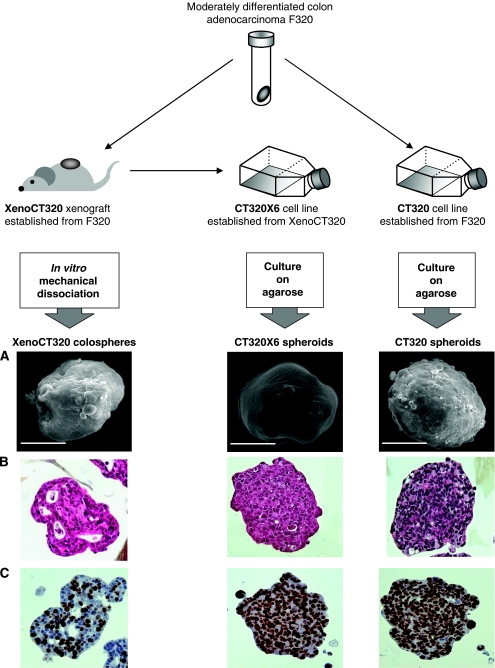
Experimental protocol leading to the production of XenoCT320 colospheres and paired spheroids of CT320X6 and CT320 cell lines. Colospheres (left panel), CT320X6 spheroids (middle panel) and CT320 spheroids (right panel) underwent SEM. (**A**) Bar=100 *μ*m; HES staining (**B**); anti-Ki67 immunostaining (**C**); magnification: × 20.

For a further characterisation of colospheres, the production of a large quantity of reproducible biological material was required. For this purpose, we used human colon cancer xenograft XenoCT320, previously established from a human primary tumour (Dangles-Marie *et al*, 2007). The tumour fragment F320 leading to the XenoCT320 establishment came from a patient who presented synchronous liver metastasis. In nude mice, axiliar lymph nodes were tumour positive when collected after the removal of the tumour xenograft and recurrence at the original engraftment site was observed (data not shown). Similar to the parent colon cancer specimen, which was included in the former clinical study, XenoCT320 tissue generated colospheres after *ex vivo* mechanical dissociation. Furthermore, the morphological features of colospheres are close to those of spheroids generated *in vitro* using permanent carcinoma cell lines. This prompted us to compare XenoCT320 colospheres with spheroids derived from the CT320X6 cell line, previously established from the XenoCT320 tumour, and with spheroids derived from the CT320 cell line, established from the same original patient tumour fragment as the xenograft (Figure 3).

**Figure 4:**
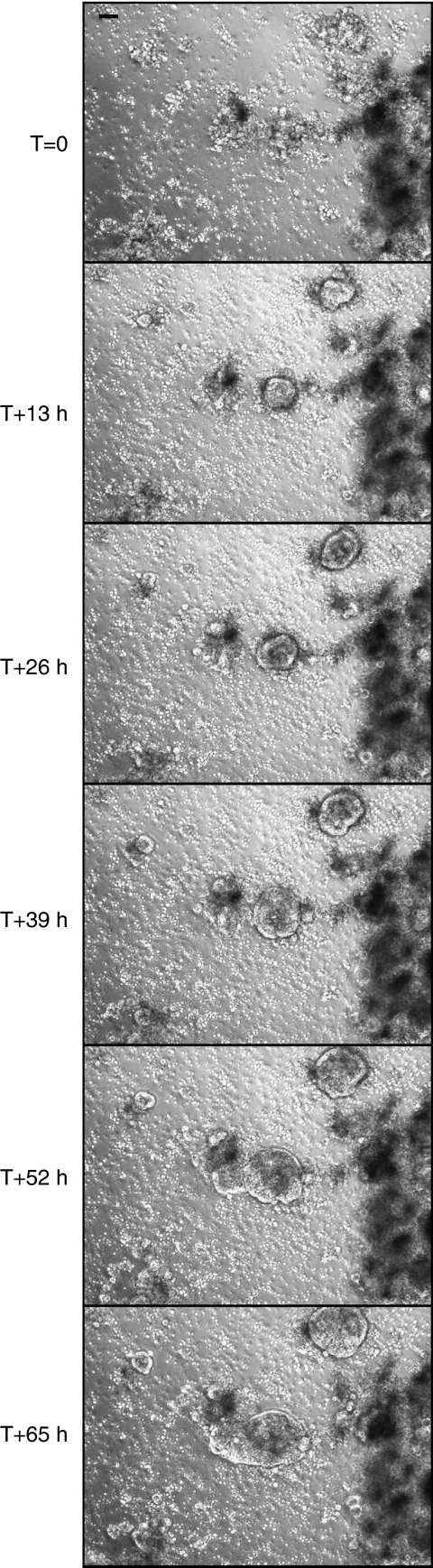
Genesis of XenoCT320 colospheres. Pictures, taken as representative of four independent experiments, at T=0 after tumour tissue dissociation, T+13, T+26, T+39, T+52 and T+65 h. Bar=100 *μ*m.

To analyse the dynamic nature of cellular events leading to colosphere formation in only 1 day, dissociated XenoCT320 tissue was filmed as a 2D outline every 4 min for 65 h (Figure 4 and Supplementary Video 1). This real-time monitoring clearly showed that large colospheres (>50 *μ*m) were generated by remodelling of tissue fragments, whereas smaller colospheres were spontaneously formed by the aggregation of single cells. Interestingly, the rapid generation of colospheres was not because of the presence of FCS, as similar experiments performed in FCS-free medium led to the same observation (data not shown).

Morphology studies were further investigated by SEM experiments that confirmed the high degree of compaction of these well-rounded structures (Figure 3A). Moreover, the outer surface of both colospheres and spheroids appeared quite smooth, in contrast to the thick filament network of extracellular matrix components entirely covering the surface of spheroids described for the human colon carcinoma HT29 cell line (Santini and Rainaldi, 1999). Using histological examination (Figure 3B), only cancer cells were observed in XenoCT320 colospheres. This observation was confirmed by cytometry analysis performed with anti-epithelial-specific human antigen EpCAM, which showed a consistent staining of all colosphere-forming cells. Real-time quantitative RT–PCR using primers specific for mouse *Tbp* confirmed the absence of mouse cells in human XenoCT320 colospheres, whereas this approach always showed the presence of mouse (stromal) cells in human XenoCT320 tumour tissue (rate<10%). Although XenoCT320 colospheres mimicked adenocarcinoma, CT320X6 and CT320 spheroids resembled only carcinoma morphology. In addition, Ki-67 staining showed that colospheres were made up of viable proliferating cells. Viability assays confirmed that colospheres remained alive for up to a minimum of 3 weeks when transferred on agarose to avoid adherence to substrate (Supplementary Figure S1). However, the latter cells proliferated less strongly than did cells forming spheroids (Figure 3C).

### Migration and invasion properties of colospheres

**Figure 5:**
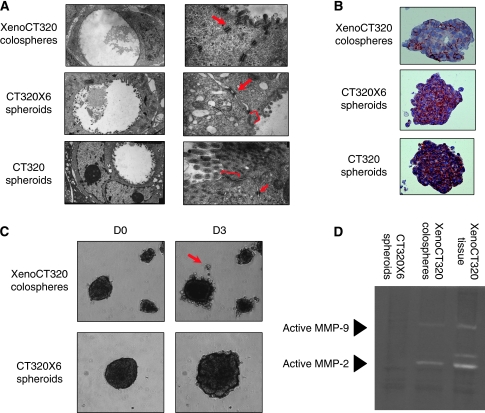
*In vitro* invasion and migration properties of XenoCT320 colospheres and CT320X6 and CT320 spheroids. These results are from one experiment representative of at least three independent experiments. (**A**) TEM experiments show acini with microvillosities (left panel), desmosomes (arrows) and zonula occludens (bracket). (**B**) Anti-E-cadherin immunostaining. Magnification: × 20. (**C**) XenoCT320 colospheres and CT320X6 spheroids were embedded in Matrigel. Cultures were photographed at days 0 and 3 after embedding. Single-cell motility could be observed with colospheres (red arrow). (**D**) Results from gelatin zymography. Detection of MMP-2 and MMP-9 activity in lysates of XenoCT320 tissue and in derived colospheres but not in CT320X6 spheroids.

As the above results from 203 patient primary tumours showed a correlation between aggressiveness of the tumour and its ability to form numerous colospheres, we next investigated the migratory and invasive properties of colospheres. Carcinoma invasiveness is often characterised by an epithelial-to-mesenchymal transition (EMT)-like dedifferentiation of tumour cells, a process by which epithelial cell layers lose polarity and cell–cell contact and undergo remodelling of the cytoskeleton, leading to a migratory phenotype (Bates and Mercurio, 2005). The nature of the cellular junction and the expression of E-cadherin, a caretaker of EMT, were thus investigated in colospheres and spheroids. Using electron microscopy, glandular structures with mucus, microvilli and desmosomes were found both in spheroids and colospheres, indicating a gut epithelial origin (Figure 5A). Nevertheless, zonula occludens, signs of tight junctions, were observed in spheroids but not in colospheres. E-cadherin immunostaining showed that membrane expression was more disturbed in colospheres than in spheroids (Figure 5B).

For functional analysis, colospheres and spheroids were embedded in Matrigel in the absence of a chemoattractant. Spherical multicellular cell aggregates were then imaged during 3 days in culture. Although colospheres and spheroids were shown above to be compacted structures using SEM, it was observed that single cells succeeded in detaching from colospheres in the Matrigel assay (Figure 5C and Supplementary Figure S2A). As for spheroids, even if larger size of spheroid clusters was shown, no isolated cell could be observed (Figure 5C and Supplementary Figure S2B).

Movement into Matrigel requires extracellular matrix degradation. Two major matrix metalloproteinases (MMPs) in colon cancer, MMP-2 and MMP-9, were then studied using gelatin zymography. Although protein extracts of spheroids displayed no gelatinase activity, colospheres and xenograft exhibited both MMP-2 and MMP-9 activity (Figure 5D).

**Figure 6:**
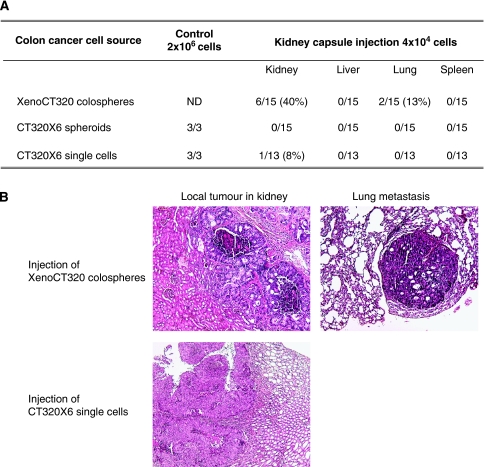
Tumorigenic and metastatic phenotype of XenoCT320 colospheres. (**A**) Injections of a quantity equivalent to 4 × 10^4^ cells as colospheres, spheroids or single-cell suspension were administered in a subrenal capsule assay in nude mice. After 14 weeks, mice were examined for local and distant tumour development. Mice were considered negative if no tumour tissue was identified either by necropsy or by histological examination. As control, 2 × 10^6^ cells were subcutaneously injected. (**B**) A well-differentiated colon adenocarcinoma was developed at the kidney site and in the lung after XenoCT320 colosphere injection under renal capsule. A poorly differentiated colon adenocarcinoma was obtained in the kidney of one mouse after injection of CT320X6 single cells. HES staining, magnification × 5.

We then tested the biological features and metastatic characteristics of colospheres through the subrenal capsule implantation of a low and equivalent number of human colon cancer cells (4 × 10^4^) injected as XenoCT320 colospheres, as CT320X6 spheroids or as a cell suspension from the CT320X6 cell line (Figure 6A). The subcutaneous injection of a large number of CT320X6 cells (2 × 10^6^ cells) as single cells or spheroids proved that this cell line was tumorigenic in nude mice. Tumour formation at the injection site occurred in 6 out of 15 mice injected with XenoCT320 colospheres. Conversely, CT320X6 spheroids formed no tumour (0 out of 15) and in the CT320X6 single-cell suspension, only one local tumour was formed in 1 out of 13 cases. In addition, a gross and histological examination of potential organ distant sites revealed the unique tumorigenic and metastatic phenotype of the colospheres: metastases were only observed in the case of colosphere injection (Figure 6B).

### Expression of colon cancer stem cell markers by colospheres

Floating spherical cell cluster cultures, such as colon cancer spheres, mammospheres and neurospheres, have been described as being valuable models for cancer stem cell culture. The round compact morphology of colospheres resembles that of colon cancer spheres. This observation and the fact that colospheres are more efficient than spheroids in the initiation of tumour growth and metastasis in immunodeficient mice led us to study the membrane expression of two cancer stem cell markers, CD133 and CD44, in XenoCT320 colospheres. We then compared this expression with that of human cancer cells in XenoCT320 tissue and with that of CT320X6 and CT320 spheroids (Table 1).

Colospheres, spheroids and carcinoma cells in xenograft highly expressed CD133. Even if the variability of the CD133^+^ cell number was high in colospheres and xenografts according to SEM, the profile of this cell sub-population was similar in parental xenograft and derived colospheres. In contrast to CD133, CD44 was expressed to a weaker extent in human cancer cells in xenograft tissue and colospheres than in spheroids. All CD44^+^ cells were CD133^+^ in colospheres and xenografts, whereas a sub-population of CD44^+^/CD133^−^ cells was present in spheroids. Furthermore, the CD133 and CD44 phenotype of colospheres remained stable even after 21 days of *ex vivo* culture (Flow cytometry data: 47.9%±14 CD133^+^ cells, 2.1%±1.8 CD44+ cells, at D21, three independent experiments).

## Discussion

This study reports the identification of colospheres, a newly characterised 3D cancer cell model directly obtained from dissociated human CRC tissue and associated with tumour aggressiveness. This model differs first from classical spheroids in that colospheres are obtained after short-term culture and not with permanent cell lines and, second, in that colospheres and spheroids clearly display distinct phenotype features.

The first step of this work was based on a large series of primary colon tumour specimens collected from 203 patients. After mechanical disruption, 98 tumours led to colospheres. A striking point is that the capacity to generate colospheres is not an intrinsic property of colon mucosa, which is not capable of generating colospheres. In contrast, only tumour epithelial cells are able to aggregate in a compact way to lead to spherical well-organised structures. The observation of such organoid structures has been reported by a limited number of studies aimed at establishing *in vitro* human colon cancer cell lines (McBain *et al*, 1984; Park *et al*, 1987; Oh *et al*, 1999) or in culture from glioma and bladder cancer biopsy specimens (Bjerkvig *et al*, 1992), but have never been studied further. We showed that the capacity to form colospheres was related to aggressiveness of the tumour, as defined by its AJCC stage. Indeed, tumours displaying local lymph node metastasis (stage III) or distant metastasis (stage IV) are more susceptible to form colospheres than are stage I and II tumours.

As colospheres are exclusively formed by cancer cells, they represent a 3D *ex vivo* culture model clearly distinct from the ‘tumour fragment spheroids’ model that consists of a variable mixture of cancer and stromal cells, obtained using the long-term cultivation of tumour biopsy specimens in an organotypic tissue manner (Wibe *et al*, 1984; Fjellbirkeland *et al*, 1995). The histological features of colospheres, including high compaction, numerous mitoses and nucleolar atypia, are close to those of both microtumours observed upon cytological analysis of carcinoma ascites and to a lesser extent to those of poorly differentiated spheroids generated with permanent carcinoma cell lines (Santini and Rainaldi, 1999).

To further characterise these colospheres, a large quantity of reproducible biological material was required. To this aim, we used a model based on a human colon cancer xenograft (XenoCT320), able to generate colospheres after *ex vivo* dissociation, and the derived colon cancer cell lines, CT320 and CT320X6, able to form compact spheroids on agarose. Similarly, we used another pair of xenograft and corresponding cell lines established in the laboratory: the XenoCT329 xenograft established from a human primary colon tumour sample in nude mice, the CT329 cell line established directly from the tumour patient and the CT329X12 cell line derived from XenoCT329 (Dangles-Marie *et al*, 2007). Colospheres were collected from dissociated XenoCT329 tissue and compared with CT329 and CT329X12 spheroids (data not shown), with comparable results with the CT320 model. This study model provided a unique opportunity to compare colospheres from dissociated CRC xenograft tissue with a conventional 3D multicellular spheroid model, derived from the same tumour specimen. It is noteworthy that the remarkable degree of organisation of colospheres was not dependent on either an exogenous basement membrane or culture within a 3D matrix, unlike the *in vitro* spheroid tumour model. Indeed, all dissociation cultures of CRC primary tumours leading here to colospheres were carried out in tissue-culture-treated flasks.

Comparison of morphological structures in colospheres and spheroids in our study model revealed that, despite their compaction, colospheres did not display tight junctions. They also displayed a disturbed expression of E-cadherin, one of the caretakers of the epithelial phenotype, compared with spheroids. Although the role of EMT in carcinoma dissemination is controversial (Christiansen and Rajasekaran, 2006), downregulation of E-cadherin has been linked to invasion and metastasis in CRC (Bates and Mercurio, 2005). These observations suggest that colosphere-forming cells maintain EMT potential. The putative involvement of these cells in cancer spread was underlined by the colospheres’ ability to migrate into Matrigel. Indeed, one of the first events that metastatic cells have to complete is the local invasion of stroma, a process that requires a degradation of extracellular matrix components by proteolytic enzymes including MMPs (Fidler, 2002; Pantel and Brakenhoff, 2004). One particular group of MMPs, the gelatinases A and B, also known as MMP-2 and MMP-9, are of particular interest with respect to the development and progression of CRC (Garbett *et al*, 1999; Mook *et al*, 2004). Gelatinase analysis showed that *ex vivo* colospheres displayed MMP-2 and MMP-9 activity, as did parental xenograft tissue, whereas paired cell line spheroids were devoid of such proteases. More importantly, as shown by a subrenal capsule assay, colospheres displayed undoubtedly a high tumorigenicity and metastatic potential, compared with the spheroid model, suggesting that these 3D structures contained more cancer-initiating cells. It could be hypothesised that the different tumorigenicity is related to a higher passage number into mice for xenografts giving rise to colospheres than for xenografts leading to the CT320X6 cell line. Nevertheless, the presumed selection of more aggressive cells through *in vivo* passage is not so clear. We have determined that the CT320 cell line, established directly from a patient sample without any passage into mice, and the CT320X6 cell line, derived from the xenograft XenoCT320, display a similar growth curve in nude mice (unpublished data). It is also noteworthy that tumours obtained after injection of colospheres were well-differentiated adenocarcinomas, whereas tumours derived from a cell-suspension injection were poorly differentiated carcinomas, illustrating, as largely observed (personal communication, Heiner Fiebig), that cancer cell lines established from differentiated human tumours lead to poorly differentiated tumours, whereas xenografts directly from tissue tumour specimens maintain their differentiation level. The differences observed between colospheres and spheroids have to be put in line with the different growth conditions (*ex vivo vs in vitro*), which in turn involve different microenvironments. The impact of the microenvironment – such as cell–cell interactions and extracellular matrix – on cell phenotype is indeed well described (Santini and Rainaldi, 1999; Jacks and Weinberg, 2002; O’Brien *et al*, 2002; Smalley *et al*, 2006; Friedrich *et al*, 2007).

Owing to their round compact architecture and their direct CRC tissue origin, colospheres can be compared with colon cancer spheres, a model previously described for colon cancer stem cell expansion cultures (Ricci-Vitiani *et al*, 2007; Todaro *et al*, 2008; Vermeulen *et al*, 2008). However, methods for obtaining colospheres and colon cancer spheres clearly differ. Indeed, colospheres are formed after a simple mechanical dissociation protocol, preserving small pieces in cultured tumour bulk, whereas colon cancer spheres are generated after an enzymatic treatment leading to a single-cell suspension. In addition, colon cancer spheres require ultra-low-attachment conditions, epidermal growth factor and fibroblast growth factor-2, plus culture for 4 weeks, whereas colospheres form in 1 day, without adding exogenous growth factors. Furthermore, these two models also differ by the expression profile of putative colon cancer stem cell markers. CD133 has been reported to be a presumed colon cancer stem cell marker (O’Brien *et al*, 2007; Ricci-Vitiani *et al*, 2007; Todaro *et al*, 2008), even if this function is now challenged (LaBarge and Bissell, 2008; Shmelkov *et al*, 2008), and CD44 has been more convincingly described as an informative marker of colon cancer stem cells in both primary tumours and xenografts (Dalerba *et al*, 2007; Subramaniam *et al*, 2007; Du *et al*, 2008). Although cells grown as undifferentiated colon cancer spheres have been reported to be exclusively CD133^+^ (Ricci-Vitiani *et al*, 2007), a large number of cells from colospheres expressed CD133, but a negative population was also present. These results are consistent with a recent study describing a variable pattern of CRC tumours that were negative to highly positive for CD133 expression (Dalerba *et al*, 2007). Likewise, the rates of CD44^+^ human cells in the xenograft and the fact that CD44^+^ cells were a minority sub-population of CD133^+^ cells are consistently in agreement with previous observations by Clarke and Coll (Dalerba *et al*, 2007). Therefore, colospheres closely reproduce the CD44 and CD133 expression profiles found in the parental xenograft.

In conclusion, these results show that colospheres form an *ex vivo* three-dimensional model distinct from traditional spheroids. It is noteworthy that colospheres are formed from a random dissociation of fragments of tumours, leading to potential variability within resulting colospheres. In contrast, spheroids are obtained from cell lines using the liquid overlay technique in microwells, giving rise to a population of identical spheroids. This heterogeneity in the colosphere population could reflect inherent intratumoral heterogeneity. As a short-term culture model associated with tumour aggressiveness, it might therefore be relevant for studying metastatic processes.

## Figures and Tables

**Table 1:** Expression of CD133 and CD44 in XenoCT320 xenograft, derived colospheres and CT320X6 and CT320 spheroids

	**Percentage of positive cells**	
	**CD133**	**CD44**
XenoCT320 colospheres	64.5±21	4.4±2.3
XenoCT320 tissue^a^	57.5±19	5.7±6.8
CT320X6 spheroids	13±2	32±2
CT320 spheroids	25±11	32.3±6.8

Data are means±s.e.m. of results from experiments carried out at least three times.

a Data are given for EpCAM^+^ cells sorted from XenoCT320 tissue.

## Supplementary Material

Supplementary Figure S2A

Supplementary Figure S2B
