# Supplementary material for: Newly characterised ex vivo colospheres as a three-dimensional colon cancer cell model of tumour aggressiveness
Source: Br J Cancer. 2009 Jul 14;101(3):473–82. doi: 10.1038/sj.bjc.6605173 (PMC2720229; doi:10.1038/sj.bjc.6605173)
Supplement: Supplementary Figure S1 [file 6605173x4.doc]

**Supplementary data legends**

**Supplementary Figure S1.** Viability of colospheres. Cell viability was evaluated by the WST-1 assay (Roche Diagnostics, Meylan, France) according to the manufacturer’s instructions. Colospheres were collected at D3 after XenoCT320 tissue dissociation and 10 colospheres per microwell were transferred on agarose in 150 µL of culture medium. Medium was changed twice a week (50 µL). At D3, D5, D10, D17, D20 and D25 after tissue dissociation, 15 µL of the WST-1 reagent were added in 12 microwells of each time-point and plates were incubated overnight before absorbance measurement. Relative cell viability was defined as the ratio of absorption measured in colospheres at different times compared with the absorption measured in colospheres at D3. The experiment was performed five times with identical results. Shown is the average data from a representative experiment ± s.e.m..

**Supplementary Figure S2.** *In vitro* invasion and migration properties of colospheres and spheroids. XenoCT320 colospheres (**A**) and CT320X6 spheroids (**B**) were embedded in Matrigel as previously described in Material and Methods and invasion/migration was monitored by time-lapse video microscopy for 70 h at an interval of 2 h. Dynamic sequences were obtained on a DM IRBE stand equipped with a motorized stage (Leica, Mannheim, Germany) using a 37°C 8% CO_2_ humidified stage-top incubator (Life Imaging Services, Switzerland). Black arrows indicate detaching cells. Scale bar = 100 µm. Time presented as hr:min.

**Supplementary video 1.** Genesis of XenoCT320 colospheres. Colosphere formation was monitored by time-lapse video microscopy for 65 h at a 4 min interval. Dynamic sequences were obtained on a DM IRBE stand equipped with a motorized stage (Leica) using a 37°C 8% CO_2_ humidified stage-top incubator as described in the Materials and Methods section. This movie, taken as representative of 4 independent experiments, shows both remodelling of tissue fragments and cell aggregation. Time presented as hr:min.
