# Supplementary material for: Newly characterised ex vivo colospheres as a three-dimensional colon cancer cell model of tumour aggressiveness
Source: Br J Cancer. 2009 Jul 14;101(3):473–82. doi: 10.1038/sj.bjc.6605173 (PMC2720229; doi:10.1038/sj.bjc.6605173)

Supplementary Figure S2 Weiswald LB *et al*

A

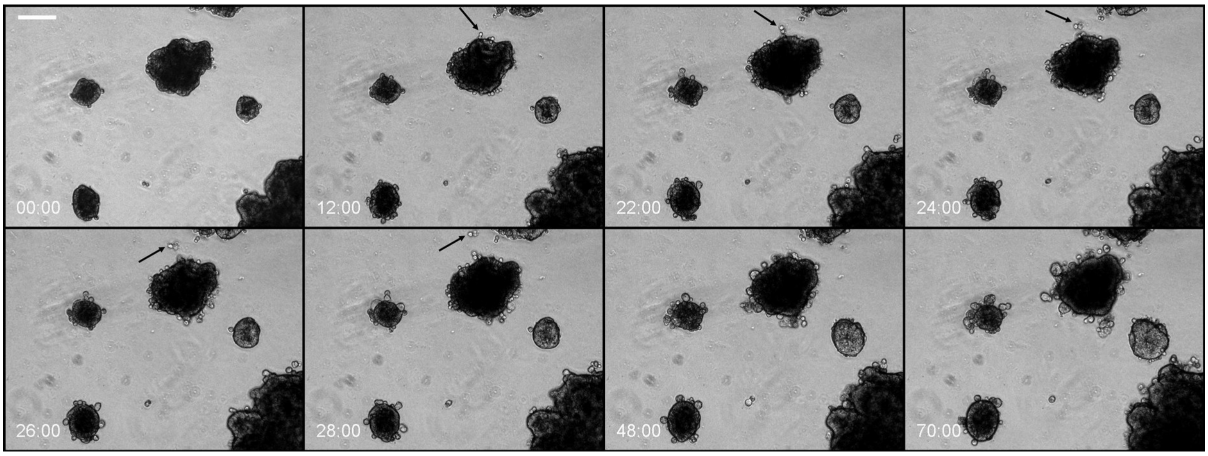

B

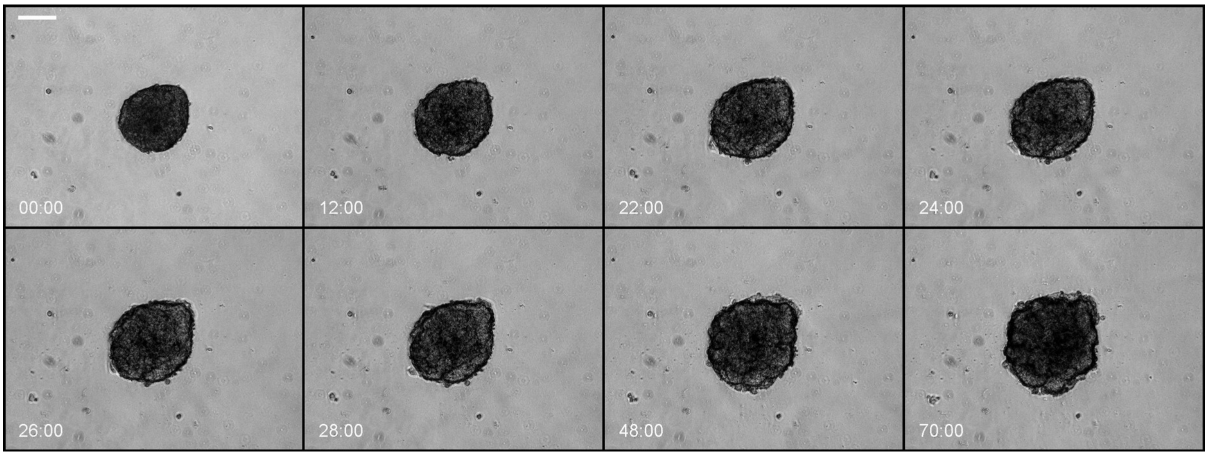

Supplement: Supplementary file 3 [file 6605173x2.doc]
